# ‘We are the advocates for the babies’ - understanding interactions between patients and health care providers during the prevention of mother-to-child transmission of HIV in South Africa: a qualitative study

**DOI:** 10.1080/16549716.2019.1630100

**Published:** 2019-07-10

**Authors:** Fiona Heerink, Anja Krumeich, Frans Feron, Ameena Goga

**Affiliations:** a Department of Social Medicine, Maastricht University, Maastricht, The Netherlands; b Department of Health, Ethics and Society, Maastricht University, Maastricht, the Netherlands; c Department of Paediatrics, University of Pretoria, Pretoria, South Africa; d Health Systems Research Unit, South African Medical Research Council, Pretoria, South Africa; e HIV Prevention Research Unit, South African Medical Research Council, Durban, South Africa

**Keywords:** Respectful maternity care, access to health care, quality of care, interpersonal interactions, personalised health care, patient-centred care, individualised care, HIV, prevention

## Abstract

**Background**: HIV/AIDS has had a significant impact on maternal and child health in South Africa. It is thus of vital importance to implement interventions to prevent mother-to-child transmission of HIV (PMTCT) as early as possible during pregnancy. Negative interactions between patients and health care providers (HCPs) can be an important barrier to antenatal care, PMTCT use and PMTCT adherence. Research about respectful maternity care has focused more on the patient perspective. We therefore compared the patient and HCP perspectives and reflected on how interactions between HCPs and patients can be improved.

**Objective**: To obtain insights into the attitudes of HCPs in the context of HIV and PMTCT-related care, by studying patient and HCP perceptions of their interactions, in a peri-urban hospital setting in Gauteng province, South Africa.

**Methods**: A qualitative study was conducted in a public tertiary-level hospital. Fourteen semi-structured in-depth interviews were conducted with nurses and doctors in the antenatal clinic and postnatal ward. Thirty-one semi-structured in-depth interviews and two focus group discussions were conducted with HIV positive and negative women on the postnatal ward.

**Results**: HCPs experienced a difficult work environment due to a high workload. This was combined with frustrations when they felt that patients did not take responsibility for their own or their child’s health. They were motivated by the need to help the child. Patients experienced judging comments by HCPs especially towards younger, older and foreign women. They expressed fear to ask questions and self-blame, which in some cases delayed health care seeking. No discrimination or isolation of HIV infected patients was reported by patients and HCPs.

**Conclusion**: We hypothesize that more humane working conditions for obstetric HCPs and a caring, personalised approach to patient management can improve patient-provider interactions and access to respectful care. These are critical to preventing mother-to-child transmission of HIV.

## Background

HIV-related care is critical for optimal maternal and child health (MCH) in high HIV prevalence settings such as South Africa. Non-pregnancy related infection, mainly HIV-related, is the largest contributor (40.5%) to maternal mortality in South Africa [1]. Furthermore, HIV/AIDS has impeded South Africa’s ability to timeously meet the fourth millennium development goal of reducing child mortality [2]. However, in the last five to ten years South Africa has made substantial progress in MCH, partly due to the scale-up of interventions that prevent HIV transmission from mother-to-child (PMTCT) [2].

PMTCT interventions in South Africa are integrated into basic maternity care [3]. PMTCT interventions include HIV testing and counselling in pregnant women during antenatal and postnatal care, strengthening retention in care and adherence to triple antiretroviral therapy (ART), and providing appropriate treatment, care and support [4]. In 2011, national policy in South Africa changed to support exclusive breastfeeding until 6 months with continued breastfeeding until 12 months, regardless of maternal HIV status [3,4]. Research in South Africa illustrated that antenatal intention not to breastfeed and indecision about feeding choice were most strongly associated with breastfeeding cessation 12 weeks postpartum [5]. It is thus important to reach HIV-positive women in early pregnancy to initiate PMTCT interventions.

Previous research in South Africa and Sub-Saharan Africa has demonstrated that access to maternity care is not only affected by barriers such as availability and affordability; interactions between health care providers (HCPs) and patients are also important factors [6–16]. Additionally, negative treatment by HCPs can be an important barrier to PMTCT adherence [9,11]. Several categories of disrespectful care by HCPs have been identified in South Africa, including substandard care and negligence, poor communication with patients and non-consented care, physical and verbal abuse, and services in exchange for bribes [14]. Respectful maternity care is defined as ‘an approach centred on the individual, based on principles of ethics and respect for human rights, promoting evidence-based practices that recognize women’s preferences and women’s and new-born’s needs’ [17] and includes care before, during and after childbirth [18,19].

Several factors influence the provision of respectful maternity care by HCPs, including staff shortages or high workload, long working hours, and lack of equipment [8,9,12,17,20]. However, some HCPs report that lack of empathy may be a coping strategy as humanizing patients’ emotions could make HCPs more susceptible to burn-out [21]. HCPs also report feeling frustrated when patients do not follow instructions, whilst others believe that HCPs have power and control over patients and are better, as they are usually from a higher socioeconomic class compared with patients [9,17,22]. Notwithstanding these, disrespectful care results in patient fear and could delay maternity care seeking [6,7,11,12,14,19]. Women may even choose home delivery or a traditional birth attendant instead [12,22–25].

Research about respectful maternity care and access to health care has focused mainly on the patient perspective [12,22]. To obtain insight into the attitudes of HCPs in the context of PMTCT-related care, we studied patients’ and HCPs’ perceptions of their interactions. We compare both perspectives on the problem definition, underlying assumptions and potential solutions. The latter (perspectives on solutions) will fill a gap in current literature as there are few published studies on factors promoting positive attitudes and behaviours among HCPs.

## Methods

This qualitative study was inspired by Bacchi’s social constructivist approach, namely ‘What is the problem represented to be? (WPR)’ [26]. Bacchi highlights that the representation or definition of a problem differs from one stakeholder (patient) to another (HCP) and that these differences are rooted in personal and professional backgrounds. Consequently, this approach focuses on four important questions, namely: ‘What presuppositions or assumptions underlie the problem definitions of the different stakeholders?’; ‘What dimensions of the problem are left unproblematic in each of these problem definitions?’; ‘What solutions for the problem follow from the problem definition?’; and ‘What impact do these problem definitions and proposed solutions have?’ [26].

Applying the WPR-approach, we chose qualitative methods, which allow open-ended, emerging questions and probing, to develop a holistic understanding of the problem and context, based on patients’ and HCPs’ own words [27–29]. Semi-structured interviews (SSIs) were combined with focus group discussions (FGDs) for triangulation [27] and comprehensive understanding [30].

### Research population and setting

The study was conducted in a public hospital serving a poor population, in Gauteng province, South Africa. Although mainly peri-urban, the patient population includes patients from rural areas, other South African provinces and other African countries. English is the second language of most patients. A recent study that has just been accepted for publication noted a 22% HIV prevalence amongst women delivering at the hospital (personal communication, Goga A).

#### Semi-structured interviews with patients

Patients for the semi-structures interviews (SSIs) were recruited in the postnatal ward and approached by the research assistant and FH. The ward has space for up to 40 patients at a time and, on average, one patient was interviewed per day during 8 weeks of data collection. Patients were eligible, if they met all the following criteria: at least 18 years old; inpatient in postnatal ward; within the first week postpartum and not being in acute pain, emotional distress or discomfort. Both HIV-positive and HIV-negative patients were eligible for inclusion, because in this setting all patients are at risk of HIV infection and need timely access to antenatal care (ANC) to test for HIV. Patients who fitted the eligibility criteria were identified through ward records. Purposive sampling [27] was used so that key groups (such as younger women, older women) of participants were targeted. Patients were selected either with different characteristics compared to the patients who were interviewed before to test emerging theory, or with similar characteristics as previous interviewees until theoretical saturation occurred [27,28]. These characteristics included age, origin, HIV-status and delayed access to maternity care (Table 1).

**Table 1.** Characteristics of participants.

Participants	Characteristic	Categories	Frequency
Semi-structured interviews: patients	Age	18–20	6
		21–35	21
		> 35	4
	Origin	South Africa	22
		Other African Country	9
	HIV-status	Positive	10
		Negative	21
	Start antenatal care	Before 6 months gestation	18
		After 6 months gestation	7
		Unknown	6
	Home delivery	Yes	5
		No	26
Semi-structured interviews: health care providers	Age	< 30	4
		30–39	4
		40–49	3
		> 49	3
	Profession	Professional nurse	2
		Staff nurse	5
		Auxiliary nurse	4
		Medical doctor	3
	Location	Antenatal clinic	5
		Postnatal ward	9
	Work experience	< 5 years	7
		5–10 years	2
		> 10 years	3
		Unknown	2
Focus group discussions: patients	Age	18–20	2
		21–35	6
		> 35	2
	HIV-status	Positive	4
		Negative	6

#### Focus group discussions with patients

FGD questions were informed by the interviews and were conducted in weeks 3 and 5 of data collection. A mix of purposive and convenience sampling [27] was used to select FGD participants. On the day of the FGD the nurse in charge of the ward helped to identify patients that met eligibility criteria listed under SSIs, and sixteen patients were approached.

#### Semi-structure interviews with health care providers

Fourteen HCPs participated in the SSIs. HCPs who worked in the hospital were eligible if they met all the following criteria: at least 18 years old; working as a professional nurse, staff nurse, auxiliary nurse or medical doctor and working in the antenatal clinic or postnatal ward. Purposive sampling was used [27]: HCPs were targeted based on their age, profession and the location (antenatal clinic or postnatal ward) until theoretical saturation was reached. Participating HCPs were aged between 25 and 58 years and their work experience in maternity care ranged from 9 months to 36 years (Table 1).

### Data collection

To bridge cultural and language barriers during the interviews, two research assistants were employed. Both assistants attended a structured training course, conducted by FH, about qualitative data collection using the research protocol and the Qualitative Research Methods Field Guide by Mack et al. [28]. During three consecutive days they were trained in participant recruitment, moderator skills for SSIs and FGDs, and ethical considerations. FH mentored the research assistants who interviewed consenting patients in the postnatal ward. A guide consisting of eleven questions for patients and twelve questions for HCPs was used for the SSIs and FGDs (Appendix). SSIs usually lasted between 30 and 60 minutes and FGDs lasted between 55 and 80 minutes. All interviews were conducted in a private space. Interviews were only voice recorded with participant consent. 35 out of 45 SSIs and both FGDs were voice-recorded. Notes were taken during all interviews. Following each interview, FH and the interviewing assistant held a debriefing.

### Data analysis

The voice-recorded SSIs and FGDs were transcribed verbatim. One of the research assistants aided with the transcription of voice recordings. Field notes of the remaining ten SSIs and of both FGDs were elaborated in Microsoft Word. All SSIs and FGDs were analysed with thematic analysis [31] using NVivo (11) software for qualitative data analysis. Notes were taken of emerging themes during data collection and analysis.

Transcripts and notes of the interviews were first reviewed to familiarize ourselves with the data. Initially, a set of fifteen transcripts was analysed to generate initial codes using an inductive approach [27] by going through each transcript and identifying topics line-by-line. An iterative process was used to organize topics that arose from the transcripts into (sub)categories, for example ‘negligence’ under the subcategory ‘causes of HIV’ and the category ‘HCP experiences’. Coding of the transcripts and the definition of (sub)categories was discussed among the authors. An initial diagram was developed in search for relationships between categories. Using a deductive approach [27], the codes and subcategories were organised into themes based on Bacchi’s WPR-approach. The code ‘negligence’ as a cause of HIV for example, became part of the subtheme ‘patients who do not take responsibilities’ under the theme ‘problems ascribed to the patients’ and the category ‘problem definition’ as part of the HCP perspective [26].

A coding scheme was developed following discussion and was based on emerging themes. The coding scheme was used to code the complete set of SSIs and FGDs in NVivo (11). When needed, new codes and subthemes were added, and the coding scheme was refined until expert agreement on the themes was reached between the authors. The initial diagram was adjusted, guided by Bacchi’s WPR approach [26], illustrating relationships between findings. Resulting themes and subthemes are reported in this article.

### Ethical considerations

The South African Medical Research Council Ethics Committee approved the research protocol (Protocol ID: EC008-6/2014), and permission was received from the hospital research committee. All participants provided written informed consent before participating in the study. The two research assistants had a similar cultural background as patients and HCPs to ensure cultural integrity and research validity. The research assistants and FH emphasised that participants were not obligated to answers questions that caused discomfort. All interviews and data analyses were conducted confidentially and in line with the Helsinki Declaration [32].

## Results

Results from 45 SSIs (31 patient SSIs, 14 HCP SSIs) and two patient FGDs including 10 patients are synthesised below. The HCPs appeared to apply three main themes relating to their interaction with patients during PMTCT. They distinguished ‘**problems ascribed to the patients’, ‘problems ascribed to the workload’** and an overlapping theme of **‘problems ascribed to patients and the workload’**. The main theme emerging from a patient perspective related to **‘problems ascribed to health care’**. For each theme we describe a) the problem definition, b) underlying assumptions about the problem, and c) underlying assumptions about effects of the problem. For the small overlapping theme of ‘problems ascribed to the patients and the workload’ only the problem definition will be described. The results will finish with a description of possible solutions. Subthemes are italicised in the text, under each subheading.

### Health care provider perspective

#### Problems ascribed to the patients: problem definition

HCPs described how disrespectful treatment can be a patient-problem if patients do not take responsibility for their positive HIV-status. HIV was compared with other chronic diseases: *patients can live a normal life if they take good care of themselves*. Some HCPs said that it is not a problem to have a baby, providing the mother looks after herself.

‘So, these days it’s no longer people dying of HIV and AIDS.’ […] ‘If a person dies of this, it’s because of negligence. Maybe the person stopped using the drugs or the ARVs [antiretrovirals].’ (SSI participant 35, HCP)

Negligence was described by HCPs as an important underlying cause of HIV and *patients who do not take responsibility* during pregnancy and breastfeeding was a common topic. Another problem ascribed to the patients was *insufficient knowledge or wrong beliefs about HIV*, for example due to a lack of education.

Being HIV-positive and pregnant is a risk according to some HCPs. HCPs explained that *when patients do not take responsibility for their health, they expose their babies to the risk of vertical transmission of HIV*. HCPs said that they do not feel good when women put their health and their baby’s health at risk – this is unfair to the baby.

‘It also makes me a little bit angry, to know that there is an unborn child, an innocent child, that is at risk of getting HIV infected, because of adult or the mother’s mistake or neglect.’ (SSI participant 44, HCP)

#### Problems ascribed to the patients: underlying assumptions about the problem

HCPs described underlying assumptions about the responsibilities of patients and HCPs and how they handle situations when patients do not live up to the expectations. They felt that a *patient’s main responsibility is to take good care of themselves and their baby’s health*. HCPs emphasized the importance of taking medication, breastfeeding exclusively and having a healthy lifestyle. Other responsibilities of patients were to use condoms and to attend ANC during pregnancy.

‘She is positive, but it is possible for the baby to not be and she must do everything in her power to ensure that that baby does not fall, does not get infected.’ (SSI participant 44, HCP)

They also explained that *HCPs’ main responsibility is to help patients take good care of themselves and the baby*. HCPs want to treat patients and their babies in the best way possible and prevent complications. They want to treat all patients equally, with empathy and compassion. HCPs try to support, counsel, educate and encourage patients to take their medication and breastfeed exclusively.

‘I think it is firstly my responsibility as the health care worker to make the mother realise how important it is and make her fully understand what difference she can actually make.’ (SSI participant 44, HCP)

To help patients with breastfeeding, some HCPs explained how they individualise care. Some HCPs ask about returning to work, if she can express breast milk, if she is willing to breastfeed and if she can afford formula milk. One HCP asked about the support system and whether the patient disclosed her HIV status to relatives. However, there were also HCPs who said that, apart from the medical treatment of patients, they approach all women in exactly the same way.

#### Problems ascribed to the patients: underlying assumptions about effects

In practice, HCPs find themselves in a difficult position when they see *patients who do not do what the HCP says*. The HCP becomes frustrated, when patients keep acting irresponsibly, regardless of the effort by the HCP. Because HCPs know what is good for the baby, *HCPs then become the advocates of the baby*:

‘The baby cannot talk for themselves. We are the advocates for the babies. We know what’s good, what is good for the baby’ (SSI participant 35, HCP)

When a patient does not take care of herself and the baby, HCPs try to do their best to protect the baby. They try to make the patient understand the importance of protecting the baby. These attempts may include shouting or trying to scare the patient.

#### Problems ascribed to the workload: problem definition

The second problem definition of disrespectful care was described by HCPs as a workload-problem. Several causes of the high workload were described: HCPs see *many patients coming to their hospital*, there is a *lack of resources* and the hospitals are short staffed. They see *patients coming from other provinces or countries*. In some cases, there is a language barrier, resulting in longer or more frustrating patient consultations.

#### Problems ascribed to the workload: underlying assumptions about the problem

Underlying assumptions about the high workload included assumptions about foreign patients and effects of the workload. One HCP wondered why so many people come to give birth in South Africa. Others describe aspects of xenophobia in South Africa and how some foreign patients do not pay taxes and receive free health care. *Treating people from other countries was considered to be a significant drain on limited resources*.

‘I wanted to know about why? Why everything just comes to South Africa hospitals? Then we are struggling a lot and we’re short staffed.’ (SSI participant 15, HCP)

*The intention to help everyone* was expressed by some HCPs. They want to treat everyone in the best possible way and treat everyone in the hospital equally. At the same time, HCPs need to follow the protocols, do the work correctly and prevent complications.

‘You want to be good at all cost, but due to the people, who are flooding into the hospital, it’s a problem.’ (SSI participant 35, HCP)

#### Problems ascribed to the workload: underlying assumptions about effects

As a result of the high workload and the intention to help everyone, *HCPs say that they are struggling: staff get tired and burnt-out*. One HCP describes how the work needs to be rushed when the ward is full. Another HCP explains that there is a lack of resources to treat everyone in the best possible way. Frustrations and fear to be blamed for mistakes were expressed by several HCPs.

‘It’s frustrating, the short staff, a lot of patients […] and say, maybe if there will be a mistake. Then the council is going to stand on the nurses and say that you don’t do your work, but we forget that there is a lot of people.’ (SSI participant 15, HCP)

#### Problems ascribed to patients and the workload: problem definition

One theme that was discussed showed overlap between the problem definitions of patients and the workload: *when patients do not take ownership for their own illness and it leads to an increased workload for the HCP*. An HCP explained that it is frustrating when they need to do more work, because a patient does not do what is expected:

‘Especially if you are not doing what is expected of you as a patient, but as an example by not taking your medication. Then I’m feeling, I’m doing all of this, you’re just sitting watching me do all of this and then I get really frustrated.’ (SSI participant 45, HCP)

### Patient perspective

#### Problems ascribed to health care: problem definition

*Patients often heard about negative experiences with maternity care from others*. Patients themselves had both good and bad experiences: while most *patients were positive about the health care received in general, the same patients often reported negative experiences*, such as shouting by nurses. The efforts of HCPs to encourage patients to give exclusive breastfeeding were confirmed by patients.

‘I was actually surprised, because I was expecting the worst, you see, but so caring, so understanding, you know, helpful. You ask questions, you get your answer.’ (SSI participant 5, patient)

Care for specific groups was discussed, including care for younger mothers, older mothers, foreign mothers, and mothers with HIV. *There were mixed experiences with care for younger and older mothers*. Some patients reported judging undertones when HCPs were trying to teach younger women or encourage older women to use birth control.

‘They would say horrible things. They would say like “you’re still young, but you’re sleeping around” and all that, disrespecting your family.’ (SSI participant 31, patient)

*Women with HIV did not experience discrimination or isolation*. They were generally very positive about the health care they received. However, a few reported negative experiences included gossiping about an HIV-positive patient and shouting at a patient who forgot her medication.

‘Sometimes they shout if you take your medicine or if you forget your medicine at home.’ […] ‘I was shocked because I’m HIV.’ (SSI participant 37, patient)

*Mixed experiences with care for foreign patients* were reported. Some patients did not experience bad care, but others described how they were expected to pay for health services. Patients described HCPs who did not adjust their language and shouting at foreign women.

‘Because they are from the other country and like they just treat them badly because, I think, how can I say it. They don’t deserve to be here maybe.’ (SSI participant 11, patient)

#### Problems ascribed to health care: underlying assumptions about the problem

Patients had underlying assumptions about patient responsibilities and patients’ reactions when they do not do what is expected by HCPs. *Patient responsibilities included good behaviour and showing respect*. Patients said that they must listen to HCPs and adhere to the instructions of HCPs. Often patients seemed to blame themselves for problems with health care. *When patients do not do what is expected, it was described as making a mistake.*

*Patients expected HCPs to treat people well and equally*: they must have patience and be friendly. Some patients said that HCPs have the right to be rude when patients ‘make a mistake’, but other patients disapproved this behaviour by HCPs.

‘Maybe when we will try to explain something. Maybe for them like it’s a mistake, but you know why you did such a thing.’ (FGD1 participant 6, patient)

#### Problems ascribed to health care: underlying assumptions about effects

Some patients described how they *delayed seeking health care* because they feared blame. Cases were described of foreign women who were denied access to health care. Some patients explained that they are scared to seem stupid when asking questions. Patients reported being scared that the HCPs would shout: a patient with HIV experienced side effects from her medication but was afraid to raise these concerns with the nurses and another patient was scared to tell the HCPs that she forgot her medication at home. One HIV-positive patient was afraid to mention her pregnancy to the HCP and started ANC after five months:

‘I was afraid, for the nurse: “Why are you pregnant? And you tell me to use condom.”’ (SSI participant 34, patient)

### Proposed solutions

Both patients and HCPs were asked about possible solutions to mitigate problems.

#### To address problems ascribed to the patients

HCPs proffered several ideas to address the problems ascribed to patients who do not take their responsibilities. *For patients*, education on the importance of HIV testing, attending ANC and breastfeeding was recommended, using methods such as group counselling. A good *ANC system* with communication between hospitals and clinics, and routine HIV tests was suggested to find and prevent complications from early pregnancy. Education *for HCPs* about communication skills was also suggested. HCPs emphasized a respectful way of communication, including empathy for older and younger mothers.

#### To address problems ascribed to the workload

To facilitate respectful care, HCPs proposed solutions for problems ascribed to the high workload. *For the government*, they suggested building large hospitals in other locations and collaboration with other countries to prevent foreigners and people from other provinces from travelling for health care. *For the hospital*, a bigger maternity section with more staff was suggested. There should be translators or HCPs available to communicate with patients in their mother languages. Furthermore, *HCPs were advised* that all patients who are in South Africa need to be treated with respect.

#### To address problems ascribed to health care

Patients emphasized that it is important *for HCPs* to have patience and to take time for communication, to listen and to understand the patient. HCPs should encourage patients to ask questions. Foreign patients need to be treated equally and English was advised as the primary language. *For hospital managers*, patients suggested to hire HCPs who speak foreign languages. *Patients themselves* should ask questions if they do not understand the HCP. They recommended that *both HCPs and patients* should speak in a respectful way.

## Discussion

Our study highlights the perspectives of HCPs and patients regarding disrespectful treatment during antenatal and postnatal care, in the context of PMTCT. The main findings show that HCPs view the problem as a patient-problem of women who do not take responsibility to protect their child from HIV combined with the problem of their high workload. Patients defined the problem as a health-care problem as they reported negative expectations of health care, negative personal experiences including judgemental comments by HCPs and discrimination towards foreign patients. The findings suggest a cycle of interactions in which the behaviours of HCPs, who are struggling due to the workload and who become the advocates for the child, seem to contribute to patients’ negative experiences with health care. When patients delay access to health care and are afraid to ask questions, HCPs view this as a problem with ‘irresponsible patients’.

**Figure 1.**
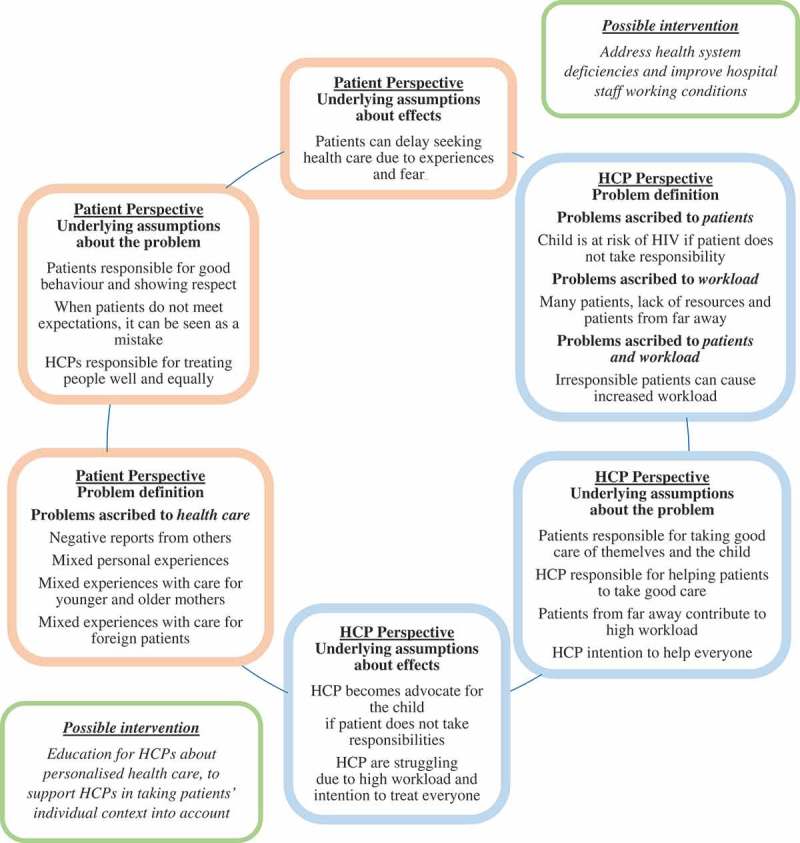
Diagram derived from our findings, inspired by Bacchi’s WPR approach [26], with two possible interventions.

Figure 1 shows a diagram in the form of a cycle with possible interactions between patient and HCP perspectives. It builds on Bacchi’s WPR approach [26], which is applied to PMTCT in this specific context in practice. The figure summarizes from both perspectives the findings (subthemes) relating to a) problem definition, b) underlying assumptions about the problem, and c) underlying assumptions about the effects of the problem. The three main themes from the HCP perspective and one main theme from the patient perspective are illustrated in the boxes ‘problem definition’. The model includes two possible interventions in italics, which will be described below. These interventions are based on the proposed solutions by patients and HCPs, the results of other studies and a reflection on the problem representations.

Bacchi’s WPR-approach looks at the effects that are produced by the problem representation [26]. For HCPs, we hypothesize that their experiences of witnessing negligence and risks to the unborn child, combined with their intention to help and perceived high workload, lead to frustrations and result in impatient behaviour and disrespectful treatment of patients. For patients, we hypothesize that fear of judgement by HCPs is a barrier to asking questions and can hinder early, timely uptake of maternity care including PMTCT related care.

Our findings, which link specifically with maternity care in the context of PMTCT show similarities with the findings of a systematic review (35 articles) which found that negative behaviours and attitudes of health care workers, poor knowledge and skills and lack of essential equipment are associated with inadequate sexual and reproductive health care services [16]. Other studies on sexual and reproductive health have highlighted similar challenges reported by HCPs, secondary to a high work load, poor skills and inability to access young women to provide appropriate preventative messages to optimise their health [15].

In our study, the high workload is attributed by some HCPs to foreign women who come to South Africa to receive health care, a phenomenon described in previous studies in South Africa [33,34]. One effect created by this problem representation is a division between foreign women and South African women. Our findings suggest that, in some cases, foreign women were excluded from free access to health care. This problem representation, however, does not consider the contextual factors that make non-South African women seek health care in South Africa.

Representing the problem as one of irresponsible patient behaviour, results in a binary of ‘responsible’ versus ‘irresponsible’ patients. Stigmatisation of patients who do not manage to live up to the expectation of HCPs was evident in the results, leading in some cases to disrespectful treatment of ‘irresponsible patients’. This problem definition makes disrespectful care a problem of patients’ individual responsibility, ignoring considerations about contextual factors such as poverty. Conversely, seeing the problem of disrespectful care as a health-care problem could make it the HCP’s individual responsibility, ignoring considerations about working in a resource-limited setting or about HCP education.

The results of our study show that it is in the interest of the child that the mother is treated respectfully. To improve respectful maternity care, it is important that good care is not only seen as an individual responsibility of patients and HCPs, but also includes a commitment from health care managers and governments [35]. In fact, an intervention conducted in 13 health facilities in Kenya demonstrated how a three-tiered intervention, targeting policy makers, HCPs (through training) and strengthening facility-community linkages, reduced disrespect and abuse during labour and delivery by 7% [35].

Based on the findings of our study and other studies [35–38] we therefore emphasize the need for a multi-level intervention targeted at improving working conditions and reducing health system deficiencies to promote HCP professional development and quality of patient care. To improve professionalization of HCPs, we recommend training on personalised, culturally and contextually appropriate care [39,40], which can be included in university curricula for medical and nursing students and in the regular in-service training for HCPs. A more personalised approach to PMTCT and maternity care will make HCPs not only an advocate for babies, but also an advocate for mothers. When all stakeholders collaborate in the common interest of MCH, early uptake of PMTCT interventions and quality maternity care may occur.

### Strengths and limitations

This research adds a model which can be used to explore, understand and improve patient-HCP interactions. The finding that HCPs act in the interest of the child has been described in literature about childbirth [12], but, as far as we know, has not clearly been described in other literature about respectful antenatal and postnatal care in the context of PMTCT. Our work has several limitations relating to the sensitive nature of the topic, the fact that it was hospital-based and that participants were generally poor with lower levels of education and language barriers. To mitigate these limitations, we employed local research assistants and held a debriefing after every interview. Patients younger than 18 years were excluded from this research study, due to the sensitive nature of topics in the interviews. However, using projective techniques, we aimed to gather some information about the experiences of younger women with maternity care. HCPs were a more homogenous group and saturation was reached at an earlier state; therefore, more patients than HCPs were included. The use of convenience sampling for the FGDs could have led to selection bias, however, the range of perspectives and topics discussed were not one-sided, so bias was unlikely. Our choice of thematic analysis has the potential limitation of reduced consistency and coherence by using research data to define themes [31] and we used Bacchi’s WPR-approach [26] to strengthen our data analysis. To ensure trustworthiness [31], we included several different perspectives to interpret our findings by including participants with varied backgrounds, crosschecking data analysis with field notes and notes of debriefing sessions with the research assistants, and having several discussions between the authors to discuss coding and themes until expert agreement was reached. Audit trails were kept, including notes of discussions between the authors [31]. Finally, due to the qualitative nature of the study, results are specific to this setting and cannot be generalized to a larger population. However, the results provide a formative understanding of interactions between HCPs and patients during maternity care in the context of PMTCT.

### Recommendations for further research

More quantitative research is needed to explain and generalize barriers to respectful care during PMTCT, especially barriers for specific groups of patients such as younger patients, older patients and patients from other countries. Therefore, we recommend testing findings from our study with quantitative questionnaires in a larger sample. Finally, our findings can be used to develop and test interventions to improve HCP-patient interactions and strengthen respectful maternity care.

## Conclusion

This research demonstrates that interactions between patients and HCPs are complex in a busy peri-urban maternity setting. The main findings are that HCPs are driven by the need to ‘do good’ for the sake of the baby, but fall into impatient behaviours because of tough working contexts. Patients reported mixed experiences with health care and are dogged by self-blame and fear. This seems to cripple the relationship between HCPs and patient. Our findings can be used to understand how interventions could affect patient-provider interactions and facilitate early booking and adherence to PMTCT related care.

## Paper context

Disrespectful care can be an important barrier to accessing maternity care. This qualitative study, conducted in a high HIV prevalence setting in South Africa, explored the perspectives of patients and health care providers about respectful care during the prevention of mother-to-child transmission of HIV. We hypothesize that more humane working conditions for medical staff, and a personalised approach to patient management, may improve patient-provider interactions and access to antenatal care, further preventing vertical HIV transmission.

## Appendix. Interview and focus group questions

***Questions for patients***

- What do you think, causes HIV/AIDS?
- What are the risks of having HIV/AIDS during pregnancy, childbirth and breastfeeding?
- What do other people think if someone they know has HIV/AIDS, e.g. a neighbour or friend, and she became pregnant?
- What would you think or what do other people think if the health care worker caring for them during pregnancy/childbirth had HIV/AIDS?
- Where did you go for check-ups during your pregnancy? How many times did you go there?
- What experiences with the obstetric health care workers in the hospital did you hear from other people, before you went there yourself?
- What is your experience with the health care workers in the hospital?
- Can you tell us about the good care that is provided for HIV positive women during pregnancy, childbirth and breastfeeding?
- Can you tell us about areas where care for HIV positive women during pregnancy, childbirth and breastfeeding can be improved?
- Do you see patients experiencing any bad care based on their age or ethnicity or HIV status? Can you tell us more about this?

***Questions for health care providers***

- What do you think, causes HIV/AIDS?
- Who is at risk for getting HIV/AIDS?
- What are the risks of having HIV/AIDS during pregnancy, childbirth and breastfeeding?
- What do other people think if someone they know has HIV/AIDS, e.g. a neighbour or friend, and she becomes pregnant?
- What would you think or what do other people think if the health care worker caring for them during pregnancy/childbirth has HIV/AIDS?
- How do you feel about practical assistance for a woman who is HIV-positive, e.g. taking blood or giving an IV, assisting with childbirth or a caesarean section?
- How would you feel if an HIV-positive woman has a complication during pregnancy or childbirth?
- How do you treat women who are HIV-positive during pregnancy, childbirth and breastfeeding?
- Can you tell us about the good care that is provided for HIV positive women during pregnancy, childbirth and breastfeeding?
- Can you tell us about the areas where care for HIV positive women during pregnancy, childbirth and breastfeeding can be improved?
- Do you think patients experience any bad care based on their age or ethnicity or HIV status? Can you tell us more about this?
- Are there any other things you would like to share with us?’

## References

[1] National Committee on Confidential Enquiries into Maternal Deaths Saving mothers 2008–2010: fifth report on the confidential enquiries into maternal deaths in South Africa. Pretoria: Department of Health Republic of South Africa; 2012.

[2] Statistics South Africa Millennium development goals: reduce child mortality 2015. Pretoria: Republic of South Africa; 2015.

[3] The National Maternity Guidelines Committee Guidelines for maternity care in South Africa: a manual for clinics, community health centres and district hospitals. Pretoria: Department of Health Republic of South Africa; 2015.

[4] National Department of Health South Africa National consolidated guidelines for the prevention of mother-to-child transmission of HIV (PMTCT) and the managment of HIV in children, adolescents and adults. Pretoria: Department of Health Republic of South Africa; 2015.

[5] Doherty T, Sanders D, Jackson D, et al Early cessation of breastfeeding amongst women in South Africa: an area needing urgent attention to improve child health. BMC Pediatr. 2012;12:105. 22827969 10.1186/1471-2431-12-105 PMC3441849

[6] Silal SP, Penn-Kekana L, Harris B, et al Exploring inequalities in access to and use of maternal health services in South Africa. BMC Health Serv Res. 2012;12:120. 22613037 10.1186/1472-6963-12-120 PMC3467180

[7] Brighton A, D’Arcy R, Kirtley S, et al Perceptions of prenatal and obstetric care in Sub-Saharan Africa. Int J Gynecol Obstet. 2013;120:224–11. 10.1016/j.ijgo.2012.09.017 23228816

[8] Amnesty International Struggle for maternal health. London (UK): Amnesty International; 2014.

[9] Gourlay A, Wringe A, Birdthistle I, et al “It is like that, we didn’t understand each other”: exploring the influence of patient-provider interactions on prevention of mother-to-child transmission of HIV service use in rural Tanzania. PLoS One. 2014;9:e106325. 25180575 10.1371/journal.pone.0106325 PMC4152246

[10] Kujawski S, Mbaruku G, Freedman LP, et al Association between disrespect and abuse during childbirth and women’s confidence in health facilities in Tanzania. Matern Child Health J. 2015;19:2243–2250. 25990843 10.1007/s10995-015-1743-9

[11] Clouse K, Schwartz S, Van Rie A, et al “What they wanted was to give birth; nothing else”: barriers to retention in option B+ HIV care among postpartum women in South Africa. J Acquir Immune Defic Syndr. 2014;67:e12–18. 24977376 10.1097/QAI.0000000000000263 PMC6686681

[12] Mannava P, Durrant K, Fisher J, et al Attitudes and behaviours of maternal health care providers in interactions with clients: a systematic review. Global Health. 2015;11:36. 26276053 10.1186/s12992-015-0117-9 PMC4537564

[13] Ishola F, Owolabi O, Filippi V. Disrespect and abuse of women during childbirth in Nigeria: A systematic review. PLoS One. 2017;12:e0174084. 28323860 10.1371/journal.pone.0174084 PMC5360318

[14] Human Rights Watch Stop making excuses: accountability for maternal health care in South Africa. New York (NY): Human Rights Watch; 2011.

[15] Jonas K, Crutzen R, Krumeich A, et al Healthcare workers’ beliefs, motivations and behaviours affecting adequate provision of sexual and reproductive healthcare services to adolescents in Cape Town, South Africa: A qualitative study. BMC Health Serv Res. 2018;18:109. 29433499 10.1186/s12913-018-2917-0 PMC5810035

[16] Jonas K, Crutzen R, van den Borne B, et al Healthcare workers’ behaviors and personal determinants associated with providing adequate sexual and reproductive healthcare services in sub-Saharan Africa: A systematic review. BMC Pregnancy Childbirth. 2017;17:86. 28288565 10.1186/s12884-017-1268-x PMC5348841

[17] Reis V, Deller B, Carr C, et al Respectful maternity care: country experiences. Washington DC: Maternal and Child Health Integrated Program; 2012.

[18] The White Ribbon Alliance Respectful maternity care: the universal rights of childbearing women. Washington DC: White Ribbon Alliance; 2011.

[19] WHO The prevention and elimination of disrespect and abuse during facility-based childbirth. Geneva: World Health Organization; 2015.

[20] Rosen HE, Lynam PF, Carr C, et al Direct observation of respectful maternity care in five countries: a cross-sectional study of health facilities in East and Southern Africa. BMC Pregnancy Childbirth. 2015;15:306. 26596353 10.1186/s12884-015-0728-4 PMC4657214

[21] Vaes J, Muratore M Defensive dehumanization in the medical practice: A cross-sectional study from a health care worker’s perspective. Br J Soc Psychol. 2013;52:180–190. 23013264 10.1111/bjso.12008

[22] Bradley S, McCourt C, Rayment J, et al Disrespectful intrapartum care during facility-based delivery in sub-Saharan Africa: A qualitative systematic review and thematic synthesis of women’s perceptions and experiences. Soc Sci Med. 2016;169:157–170. 27723514 10.1016/j.socscimed.2016.09.039

[23] Mselle LT, Moland KM, Mvungi A, et al Why give birth in health facility? Users’ and providers’ accounts of poor quality of birth care in Tanzania. BMC Health Serv Res. 2013;13:174. 23663299 10.1186/1472-6963-13-174 PMC3654954

[24] Finlayson K, Downe S Why do women not use antenatal services in low- and middle-income countries? A meta-synthesis of qualitative studies. PLoS Med. 2013;10:e1001373. 23349622 10.1371/journal.pmed.1001373 PMC3551970

[25] Bohren MA, Hunter EC, Munthe-Kaas HM, et al Facilitators and barriers to facility-based delivery in low- and middle-income countries: A qualitative evidence synthesis. Reprod Health. 2014;11:71. 25238684 10.1186/1742-4755-11-71 PMC4247708

[26] Bacchi CL Analysing policy: what’s the problem represented to be? Frenchs Forest: Pearson Australia; 2009.

[27] Matthews B, Ross L Research methods: a practical guide for the social sciences. Harlow: Pearson Education Limited; 2010.

[28] Mack N, Woodsong C, McQueen KM, et al Qualitative research methods: a data collector’s field guide. Durham (NC): Family Health International; 2005.

[29] Creswell JW Research design: qualitative, quantitative, and mixed methods approaches. Los Angeles: SAGE Publications; 2014.

[30] Lambert SD, Loiselle CG Combining individual interviews and focus groups to enhance data richness. J Adv Nurs. 2008;62:228–237. 18394035 10.1111/j.1365-2648.2007.04559.x

[31] Nowell LS, Norris JM, White DE, et al Thematic analysis: striving to meet the trustworthiness criteria. Int J Qual Methods. 2017;16:1–13.

[32] World Medical Association WMA declaration of Helsinki – ethical principles for medical research involving human subjects [Internet]. 2013 [cited 2019 313] Available from: https://www.wma.net/policies-post/wma-declaration-of-helsinki-ethical-principles-for-medical-research-involving-human-subjects/.

[33] Crush J, Chikanda A South-South medical tourism and the quest for health in Southern Africa. Soc Sci Med. 2014;124:313–320. 24973022 10.1016/j.socscimed.2014.06.025

[34] Crush J, Tawodzera G Medical xenophobia and zimbabwean migrant access to public health services in South Africa. J Ethn Migr Stud. 2014;40:655–670.

[35] Abuya T, Ndwiga C, Ritter J, et al The effect of a multi-component intervention on disrespect and abuse during childbirth in Kenya. BMC Pregnancy Childbirth. 2015;15:224. 26394616 10.1186/s12884-015-0645-6 PMC4580125

[36] White J, Phakoe M, Rispel LC “Practice what you preach”: nurses’ perspectives on the code of ethics and service pledge in five South African hospitals. Glob Health Action. 2015;8:26341. 25971398 10.3402/gha.v8.26341 PMC4430685

[37] Armstrong SJ, Rispel LC, Penn-Kekana L The activities of hospital nursing unit managers and quality of patient care in South African hospitals: a paradox? Glob Health Action. 2015;8:26243. 25971397 10.3402/gha.v8.26243 PMC4430688

[38] Sprague C, Chersich MF, Black V Health system weaknesses constrain access to PMTCT and maternal HIV services in South Africa: a qualitative enquiry. AIDS Res Ther. 2011;8:10. 21371301 10.1186/1742-6405-8-10 PMC3058008

[39] Miller S, Abalos E, Chamillard M, et al Beyond too little, too late and too much, too soon: a pathway towards evidence-based, respectful maternity care worldwide. Lancet. 2016;388:2176–2192. 27642019 10.1016/S0140-6736(16)31472-6

[40] Thomson KA, Telfer B, Awiti PO, et al Navigating the risks of prevention of mother to child transmission (PMTCT) of HIV services in Kibera, Kenya: barriers to engaging and remaining in care. PLoS One. 2018;13:e0191463. 29364979 10.1371/journal.pone.0191463 PMC5783372

